# Acute Purulent Ophthalmia Following the Suppression of an Habitual Leucorrhœa
*Nouv. Biblioth. Mars, 1829.


**Published:** 1830-01-01

**Authors:** 


### XXIII.

### ACUTE PURULENT OPHTHALMIA FOLLOWING THE SUPPRESSION OF AN HABITUAL LEUCORRHŒA.*

We have often been surprised at the reckless manner in which medical men endeavour to check habitual discharges from the vagina, by means of astringent or stimulating injections. Fortunately in most cases Nature knows what is advantageous for the patient better than the doctor, and the mischievous medication fails in accomplishing the injurious end at which its author is blindly aiming. The following case is an instructive one.

* Nouv. Biblioth. Mars, 1829.

*Case.* Madame H——, ætatis 27, of delicate and nervous constitution, and subject to mænorrhagia, had suffered for some time from leucorrhœa unattended with any irritation in the vagina or neighbouring parts. She was married, had one child four years old, and had recently experienced a miscarriage. The system being much debilitated and the leucorrhœa troublesome, Dr. Gibert, her medical attendant, ordered injections and lotions of cold water with a fourth part of vinegar of roses. The patient who was anxious to be rid of her unpleasant malady, exceeded the prescribed quantity of vinegar in the injection, and the consequence was an almost instantaneous suppression of the leucorrhœal flux. At the same time the eyes became red and painful, lacrymation was abundant, and a copious discharge took place during the night which glued together the eye-lids and stained the linen. On the 21st of June, 1828, seven or eight days after the first appearance of the ophthalmia, the conjunctiva was deeply injected, the eyelids of bright red, swelled and affected with painful smarting. Dr. Gibert now began to be alarmed, and ordered mustard poultices to the thighs, and the steam of warm water to the vulva in order to restore, if possible, the discharge; a blister to the arm, twelve leeches to the jaws, cold applications to the eyes, darkness, and the antiphlogistic regimen. It must be confessed that the Doctor shewed strict impartiality in the choice of situations for his remedies, attacking at one and the same time the eyes, the jaws, the arm, the parts of generation, and the thighs! But no matter—allons; on the two following days the pain and smarting of the eyes were exasperated, the left eye particularly was extremely red, the purulent discharge was copious, the eyelids greatly swollen. In addition to the former means pills of *cynoglosse* were given, and two porringersful of blood abstracted from the arm. On the 24th the inflammation was in some degree lessened in the left eye, but the pain and inconvenience were increased in the right. Two porringersful of blood were again taken away with the best effect, and on the 29th the catamenia re-appeared, but continued only three days instead of eight, as they had ordinarily done. The mitigation of the inflammatory symptoms in the eye "progressed," as our Transatlantic confrères would say, but so late as the thirty-second day after their first appearance the sensibility of the organ was still extreme, and the discharge very abundant and of purulent character. Narcotic and astringent collyria had been employed, but the Doctor now determined to ascertain whether that which had raised the d—l might not lay him; in fact, whether the injection which when used for the vagina had driven the disease to the eye, might not now if applied to the eye drive it back again. Accordingly a collyrium of water and vinegar of roses was employed, the discharge very quickly arrested, and the lady soon able to return to her occupations, though coloured glasses were worn for the sake of precaution. Several months afterwards Dr. Gibert had an opportunity of seeing the patient again and found that her vision was still weaker than it had been before the attack, and that the leucorrhœa had slightly returned. The case is one of much practical interest, and is calculated to inspire caution where caution is much wanted.

